# Endoscope-Assisted Surgery vs. Burr-Hole Craniostomy for the Treatment of Chronic Subdural Hematoma: A Systemic Review and Meta-Analysis

**DOI:** 10.3389/fneur.2020.540911

**Published:** 2020-11-05

**Authors:** Songyi Guo, Wei Gao, Wen Cheng, Chuansheng Liang, Anhua Wu

**Affiliations:** Department of Neurosurgery, The First Affiliated Hospital of China Medical University, Shenyang, China

**Keywords:** chronic subdural hematoma, cSDH, chronic subdural hemorrhage, endoscope, meta-analysis

## Abstract

**Objectives:** This article aims to evaluate the safety and effectiveness of endoscope-assisted surgery for chronic subdural hematoma (cSDH) in comparison with the burr-hole craniostomy.

**Methods:** An electronic literature research was performed in MEDLINE, the Cochrane library, and EMBASE from the inception to February 18, 2020. A systematic review with meta-analyses was conducted to compare the efficacy of endoscope-assisted surgery with Burr-hole Craniostomy (BHC) surgery.

**Results:** This meta-analysis included four studies comprising 441 patients. Endoscope-assisted surgery significantly decreased the risk of recurrence in patients with cSDH [odds ratio, 0.368; 95% confidence interval (CI), 0.178–0.759; *P* = 0.007; *I*^2^ = 0%]. The complication rate was also significantly lower in the endoscope-assisted group (OR, 0.249; 95% CI, 0.07–0.882; *P* = 0.031; *I*^2^ = 71.87%).

**Conclusion:** We conducted the first meta-analysis of endoscope-assisted surgery for cSDH. The meta-analysis of four studies comprising 441 patients with cSDH suggests a significantly decreased risk of recurrence and postoperative complications after endoscope-assisted surgery. Therefore, endoscope-assisted surgery is effective and safe in treating cSDH.

## Introduction

Chronic subdural hematoma (cSDH) is a common disease characterized by abnormal accumulation of blood in subdural space. Most of cSDH patients have traumatic histories, and age, male, and use of antithrombotic drugs are risk factors of cSDH. The incidence of cSDH is 13.5 per 100,000 per year, and it is five times more in people older than 65 years (1). There are several treatment strategies for cSDH, including minimally invasive surgery, such as twist drill craniostomy and burr-hole craniostomy, or relatively highly invasive surgery, craniotomy (2). However, a high recurrence rate is observed in patients undergoing twist drill and burr-hole craniostomy because of inadequate exposure of hematoma cavities during surgery. Craniotomy is more effective in evacuation of hematoma and has a lower reoperation rate than Burr-hole Craniostomy (BHC) and Twist Drill Craniostomy (TDC) (3). Nevertheless, cSDH patients are often elderly and have various medical comorbidities, which makes it hard to conduct craniotomy.

In recent years, the endoscopic surgery technique has been applied in the treatment of cSDH. The endoscope provides a broader inspection of subdural space and does less harm to patients. Several studies have proven that endoscope-assisted surgery is effective in lessening postoperative complications and recurrence rates (4–8). However, there was no large-scale and controlled study focused on this aspect. Therefore, we conducted a systemic review and meta-analysis of endoscope-assisted surgery in cSDH to explore the efficiency and safety of endoscopic surgery.

## Materials and Methods

This systematic review and meta-analysis was performed according to the PRISMA (Preferred Reporting Items for Systematic Reviews and Meta-Analysis) criteria (9).

An electronic literature research was performed in MEDLINE, the Cochrane library, and EMBASE from the inception to February 18, 2020. The terms “chronic subdural hematoma,” “refractory subdural hematoma,” “chronic subdural hemorrhage,” “refractory subdural hemorrhage,” “endoscope,” and “endoscopic” were combined to search for available studies. We also review the bibliographic list of retrieved articles to get additional related studies.

### Selection Criteria

Two authors assessed the title and abstract independently to select the eligible studies. The selection criteria were developed based on the following questions:

(1) (patients) adults with cSDH;
(2) (intervention) endoscope-assisted surgery;
(3) (comparator interventions) burr-hole craniostomy;
(4) (outcomes) recurrence, postoperative complications and mortality;
(5) (methods–study design) randomized controlled trials (RCTs) or non-RCT or retrospective or prospective controlled study;
(6) (time or duration) follow-up time longer than 6 months.

The full-text articles were then retrieved to further selection. The following studies were included (a) studies comparing outcomes of endoscopic surgery with conventional surgery, (b) RCT or a non-RCT, or a retrospective or prospective controlled study. The following articles were excluded: (a) single-arm studies that only report endoscopic surgery outcomes; (b) reviews, case reports, abstracts; (c) acute subdural hematoma or infant subdural hematoma studies.

### Data Extraction

Two researchers independently extracted the following clinical data from selected studies: basic characteristics of studies (author publication year, study design, sample size, age, and sex), clinical features of the study population (use of anticoagulation or antiplatelet drugs, unilateral or bilateral hematoma, hematoma volume, and midline shift), and clinical outcomes (recurrence rate and postoperative complication rate).

### Quality Assessment

Two reviewers conducted the assessment independently using the Newcastle–Ottawa Scale (NOS). The NOS evaluates each study according to three factors: patient selection, the comparability among groups, and the measurement of outcomes. Disagreements were settled through discussion between two researchers. If there was still no consensus, we consulted a third reviewer.

### Data Analysis

Statistical analyses were performed using RevMan 5.3 (10) and OpenMeta (11) software. Continuous variables use the mean difference as the indicator of the effect amount, and the binary variables use the odds ratio (OR). Each effect size is expressed as a 95% confidence interval (95% CI). We applied a continuity correction to all four cells if the event rates were zero. Heterogeneity tests were performed on the included studies by the χ^2^ test, and the magnitude of heterogeneity was determined by combining the *I*^2^ values. If there was no heterogeneity between the results (*P* > 0.10 and *I*^2^ ≤ 50%), then a fixed-effect model was used; otherwise, if there was heterogeneity between the results (*P* ≤ 0.10, *I*^2^ > 50%), a random-effects model was used.

### Outcome Measure

In this meta-analysis, we studied the recurrence and complications after surgery. Recurrence was defined as a reaccumulation of hematoma seen on a computed tomography scan with symptoms. Any complications or symptoms caused by cSDH surgery were defined as complications. Mortality was defined as death due to cSDH or surgery.

## Results

### Study Selection

**Figure 1:**
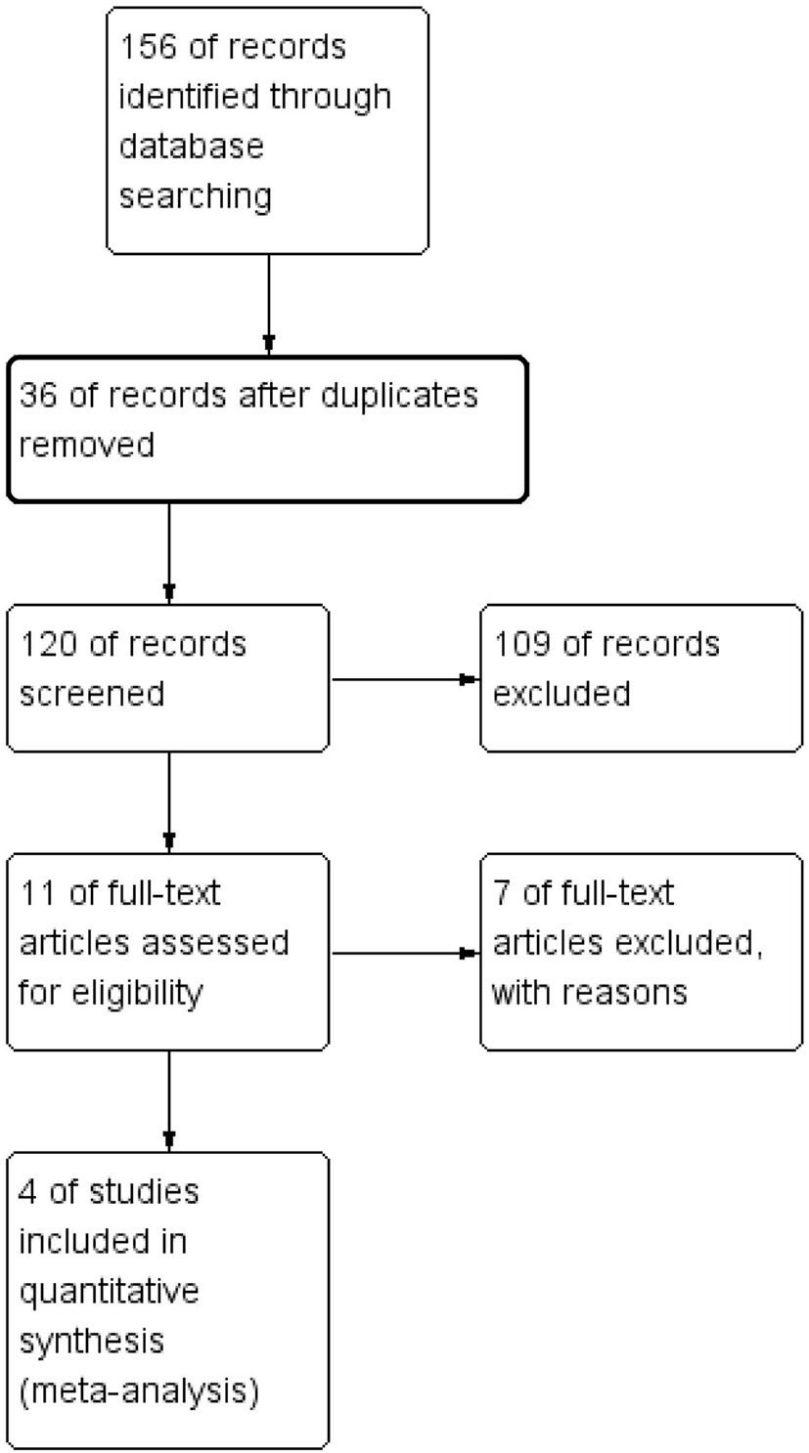
Study flow diagram.

One hundred fifty-six articles were initially generated through search in three databases. Thirty-six duplicated or nonrelated articles were excluded after examination. We then carefully reviewed the title and abstract; 109 studies were not eligible, including studies on acute SDH or infants, case reports, abstracts, and irrelevant studies. Eleven studies have remained, and their full texts were acquired for assessing their eligibility. Finally, seven single-arm studies were excluded, and four studies were eligible and included in meta-analysis (12–15). To our dismay, no RCTs were included. Figure 1 shows the search process.

### Study Characteristics

All four studies were retrospective, and 441 patients were involved, including 187 patients undergoing endoscopic surgery and 254 patients who had a burr-hole treatment. Table 1 demonstrates the baseline characteristics in both the endoscopic and BHC groups. The study by Yan did not present hematoma volume, and the study by Guan did not involve midline line shift. In each study, no statistically significant difference was found in age, sex, midline shift, hematoma volume, the proportion of bilateral hematoma, and antithrombotic drug use between the endoscopic and BHC groups. Three studies used a flexible endoscope, and one study used transparent sheath and hard neuroendoscope. All the surgeries in the four studies were successful. The follow-up time in four studies was quite different. In the studies by Du et al. (12); Zhang et al. (16), the follow-up time was 6 months, and the follow-up time was 12 months in the study by Yan. The results in these three studies are comparable. In Guan's article, the follow-up time was up to 10 years. However, each cohort's recurrence time, which is the main outcome measured in this study, was 12 months. Therefore, we believe it is comparable with the other three studies.

**Table 1:** Baseline characteristics of included studies.

**Study**	**Study design**	**Group**	**Sample size**	**Sex (Male/Female)**	**Age**	**Use of anticoagulant/antiplatelet drugs**	**Unilateral hematoma**	**Midline shift**	**Hematoma volume (mL)**
Du et al. (12)	Retrospective	Endoscope	45	29/16	73.2 ± 5.5	23 (51.1%)	38 (84.4%)	9.6 ± 3.1	96.8 ± 19.2
		BHC	49	30/19	70.6 ± 6.1	30 (61.2%)	32 (65.3%)	8.8 ± 3.8	104.3 ± 21.3
Yan et al. (14)	Retrospective	Endoscope	24	10/14	66.00 ± 6.89	7 (29.2%)	18 (75%)	11.75 ± 2.89	N
		BHC	52	37/15	66.38 ± 9.35	12 (23%)	41 (78.9%)	13.00 ± 3.77	N
Zhang et al. (13)	Retrospective	Endoscope	43	32/9	74.3 (67–91)	15 (34.9%)	42 (97.7%)	9.2 (0–19.5)	115.5 (30.3–178.2)
		BHC	27	18/9	69.8 (58–81)	13 (48%)	25 (92.6%)	8.6 (0–23.3)	109.1 (37.6–182.5)
Guan et al. (15)	Retrospective	Endoscope	75	41/34	68.3 (60–81)	N	N	N	117.3 (33.2–170.5)
		BHC	126	75/51	71.4 (61.3–87.5)	N	N	N	114.6 (35.2–157.5)

### Assessment of Risk of Bias

All four studies had five stars or more in NOS (Table 2). Therefore, they were qualified with respect to bias originating from selection, comparability of groups, and outcome evaluation.

**Table 2:** Newcastle-Ottawa Scale for assessing the quality of studies in meta-analysis.

**Study**	**Adequate definition of cases**	**Representativeness of the cases**	**Selection of controls**	**Defnition of controls**	**Comparability of cases and controls on the basis of the design or analysis**	**Ascertainment of exposure**	**Same method of ascertainment for cases and controls**	**Nonresponse rate**	**Quality score**
Du et al. (12)	☆	☆		☆	☆	☆	☆		6
Guan et al. (15)	☆			☆	☆	☆	☆	☆	6
Yan et al. (14)	☆	☆		☆	☆	☆	☆		6
Zhang et al. (13)	☆	☆		☆	☆	☆	☆		6

### Synthesized Findings

**Figure 2:**
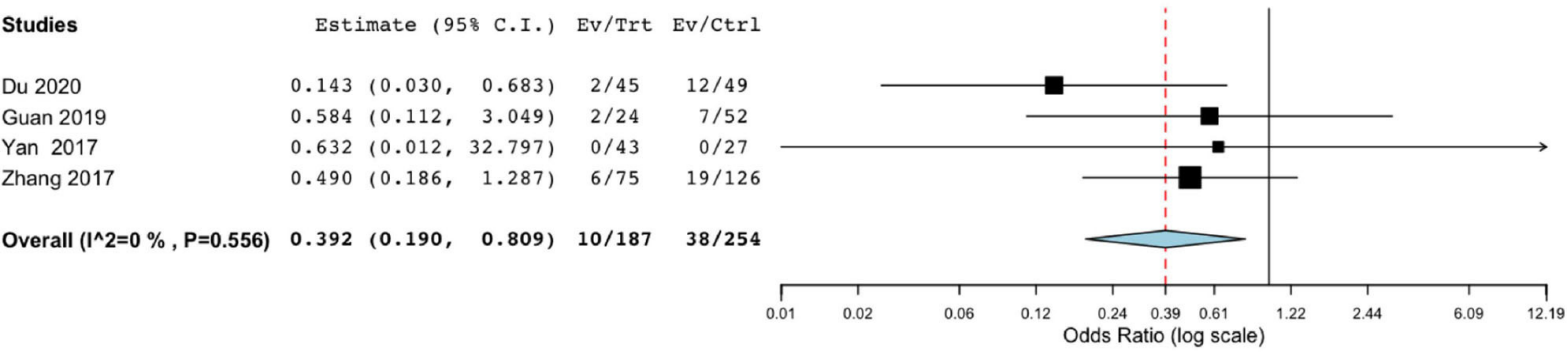
Risk of postoperative recurrence in endoscope-assisted surgery and BHC groups. (OR of 0.392, 95% CI, 0.190–0.809 , *P* = 0.011, *I*^2^ = 0%).

One thousand four hundred thirty-three patients from four studies were investigated (188 patients took endoscopic surgery, and 255 patients underwent BHC surgery, respectively). Endoscopic surgery had a significantly lower risk of hematoma recurrence in patients with cSDH (OR of 0.392; 95% CI, 0.190–0.809; *P* = 0.011, *I*^2^ = 0%; Figure 2).

**Figure 3:**
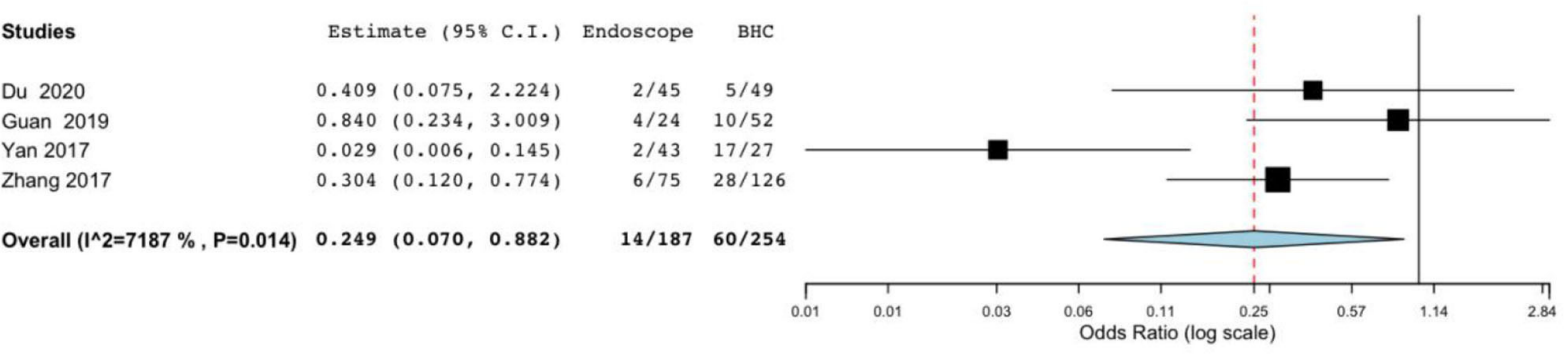
Risk of postoperative complications in endoscope-assisted surgery and BHC groups. (OR of 0.249, 95%CI, 0.07–0.882, *P* = 0.031, *I*^2^ = 71.87%).

Difference in postoperative complications was also significant in the endoscopic surgery and BHC surgery groups for cSDH (OR, 0.249; 95% CI, 0.07–0.882; *P* = 0.031; *I*^2^ = 71.87%) (Figure 3). However, heterogeneity was high, indicating the quality of evidence was relatively low.

**Figure 4:**
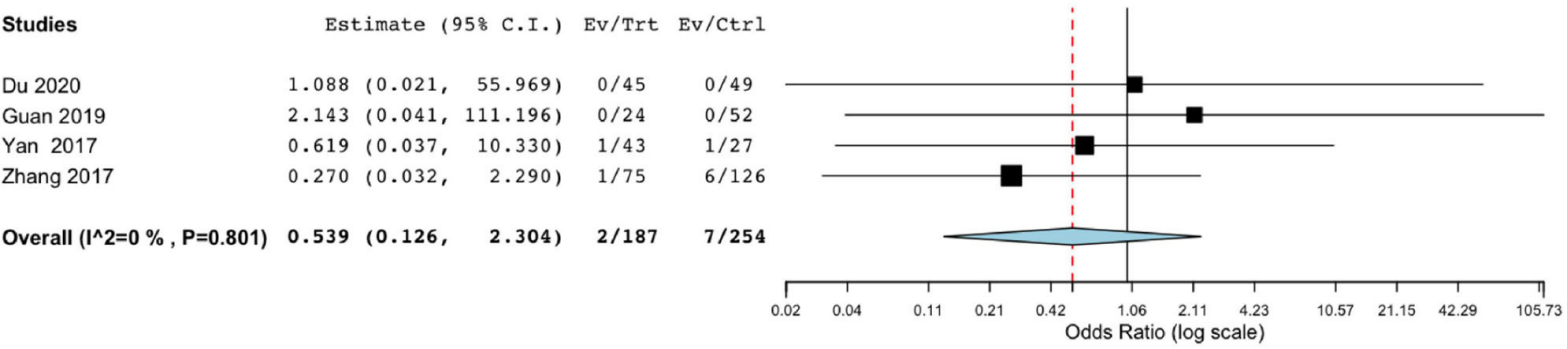
Risk of postoperative mortality in endoscope-assisted surgery and BHC groups. (OR of 0.539, 95%CI, 0.126–2.304, *P* = 0.404, *I*^2^ = 0%).

Mortality did not significantly differ between the endoscope-assisted surgery and BHC groups (OR, 0.539; 95% CI, 0.126–2.304; *P* = 0.404; *I*^2^ = 0%) (Figure 4).

## Discussion

cSDH is a common disease in neurosurgery, characterized by abnormal accumulation of blood beneath the dural layer. There are several theories that can explain the formation and progression of cSDH. Traumatic theory is the most acceptable one. It revealed that trauma is the key factor in cSDH formation; after trauma, a small and asymptomatic hematoma formed, and because of the fragility of neovasculature, microbleeding occurs and contributes to cSDH progression. In elderly people, especially in those who are older than 65 years old, their brains are naturally in atrophy state. Atrophy brain results in extension of the subarachnoid space, and the subdural veins are stretched and prolonged, which makes veins get easier to be torn and form subdural hematomas. Also, elderly people have a higher chance of falling. As a result, age is the leading risk factor for cSDH (2).

Surgery is the primary treatment for cSDH. However, it needs to be cautious for surgery on elderly patients because most elderly patients have underlying diseases, which is a restriction for highly invasive surgery. Burr-hole craniostomy is now the main method for treating cSDH, whereas many patients experienced recurrent recollection of hematoma after surgery. The postoperative blood recurrence rate has been reported to be 5–33%. It may be attributed to the inadequate clearance of hematoma, and recurrence is particularly likely to occur when there is a separation within the hematoma. The root cause is that the surgical operation is performed in a small bone hole, and the operator cannot remove the hematoma directly. Residual hematoma will impede the recovery of brain tissue, and as a result, the subdural space does not shrink, which leads to subdural gas accumulation. Residual hematomas can also cause recurrence by stimulating the secretion of inflammatory factors. These deficiencies also lead to a series of complications after surgery. The main manifestations lead to cranial hypertension, fresh bleeding, and intracranial infection.

In recent years, the endoscopic surgery technique has been applied in the treatment of cSDH. With the help of neuroendoscopy, operators can observe all corners of the hematoma cavity and confirm if there is residual hematoma. As we have known, there are many mechanisms of subdural hematoma; inflammatory factors produced by hematoma stimulation are the leading reason for the continuous expansion of hematoma (17). It is vital to confirm that there is no residual hematoma under direct observation. Also, subdural hematomas often have septa, and it is hard for BHC to clear all septa and blood during surgery. As a result, residual septa will limit the recovery of the brain and the shrinkage of the subdural space. Endoscopy can fully remove the hematoma and open the septa. Endoscopic surgery has similar or slightly larger incisions and bone holes with BHC, and local anesthesia can be used, which has little damage to the patient and is suitable for elderly patients. Although conventional craniotomy can also provide the operator with sufficient exposure and observation, the trauma caused by surgery is not proper for elderly patients.

In theory, endoscopic surgery combines the advantages of BHC and conventional craniotomy. It has a significant effect on reducing postoperative recurrence and other complications of cSDH, especially for patients who suffer recurrence or patients with septa. Our research provides literature and practical evidence and support for this concept. More direct evidence may need to be confirmed by prospective clinical studies.

In this study, we analyzed all studies comparing endoscope-assisted surgery with BHC surgery and conducted the first meta-analysis of endoscope-assisted surgery in cSDH. This article enrolled four studies involving 441 patients in the pooled analysis. All studies collected the baseline characteristics, clinical outcomes, recurrence rate, and postoperative complications of patients. Compared with BHC surgery, endoscope-assisted surgery showed a significantly low recurrence rate and complication rate. Mortality did not show a significant difference between BHC and endoscope.

In addition to the controlled studies, there are single-arm studies that also showed the capacity of endoscope-assisted surgery to improve the prognosis of cSDH patients. Moreover, a large number of studies have attempted to find a solution to decrease the recurrence rate of cSDH. Dexamethasone was used as an adjuvant treatment; however, it is demonstrated that its effect is limited in various studies (16, 18). Atorvastatin had been proved as an efficient therapy to treat cSDH in recent studies. Atorvastatin was effective in reducing the recurrence rate after surgery and promoting the rehabilitation of neurological function (19). Middle meningeal artery embolization combined with craniotomy has arisen as a promising treatment with a significantly lower recurrence rate compared to conventional surgery (20). It is also reported that inserting urokinase into the hematoma can promote clot efflux and show a better outcome.

The leading limitation of this article is the inadequately analyzed studies. No RCTs for the comparison between the endoscope-assisted surgery and the convention surgery were conducted at present. This meta-analysis is based on the double-arm observational studies. None of the evidence originates from conclusions in the gold-standard RCTs. The risk of bias is high owing to the absence of randomized allocation, and historical control groups may have a relatively weak match with the intervention group. In the future, investigators should pay more attention to endoscope-assisted surgery, and more high-quality studies and double-blind, randomized controlled clinical trials are needed to test the effectiveness of endoscope-assisted surgery in treating cSDH patients.

## Conclusion

We conducted the first meta-analysis of endoscope-assisted surgery for cSDH. The meta-analysis of four studies comprising 441 patients with cSDH suggests a significantly decreased recurrence rate and postoperative complication rate after endoscope-assisted surgery. Therefore, endoscope-assisted surgery is effective and safe in treating cSDH.

## Data Availability Statement

All datasets generated for this study are included in the article/supplementary material.

## Author Contributions

Conception and design: CL and AW. Data collection and analysis: SG, WC, and WG. Critical revision of the article: CL and AW. All authors contributed to the article and approved the submitted version.

## Conflict of Interest

The authors declare that the research was conducted in the absence of any commercial or financial relationships that could be construed as a potential conflict of interest.
